# Editorial: Subcortical pathology across dementia and motor neurodegenerative syndromes

**DOI:** 10.3389/fneur.2022.1005498

**Published:** 2022-08-25

**Authors:** Sicong Tu, Judith Machts, Michael Hornberger

**Affiliations:** ^1^Brain and Mind Centre, The University of Sydney, Sydney, NSW, Australia; ^2^Institute of Cognitive Neurology and Dementia Research, Otto-von-Guericke University, Magdeburg, Germany; ^3^Center for Behavioral Brain Sciences (CBBS), Otto-von-Guericke University, Magdeburg, Germany; ^4^Norwich Medical School, University of East Anglia, Norwich, United Kingdom

**Keywords:** neurodegeneration, neuroimaging, thalamus, Parkinson's disease, mild cognitive impairment, amyotrophic lateral sclerosis, motor neuron disease, dementia

Subcortical pathology is known to be highly prevalent in dementia and motor neurodegenerative conditions, however, how these subcortical changes impact on disease presentation, progression and symptomology is still highly debated. This is not trivial since virtually all cortical regions project or have feedback loops to subcortical structures, indicating that subcortical structures contribute to the well-known “cortical symptomology.”

Over the past decade, there have been significant advancements of *in-vivo* characterization of regional and subnuclei changes within subcortical brain structures and their respective neural connections. Increasingly, the importance of subcortical pathology is being recognized in the early and even asymptomatic stage of neurodegenerative disorders, with overlapping patterns of involvement identified with increasing disease progression.

The focus of this Research Topic was to highlight current advances in the understanding of subcortical pathology across the spectrum of dementia and motor neurodegenerative syndromes. In particular, it addresses how do these subcortical changes: (i) relate to early clinical diagnosis and phenotyping; (ii) are potential disease markers of motor, cognitive and behavioral impairment; (iii) relate to disease progression, and (iv) can be characterized using cutting-edge technologies. We present five articles highlighting the critical role of subcortical pathology across the spectrum of neurodegenerative conditions, including mild cognitive impairment (MCI), Parkinson's disease (PD), and amyotrophic lateral sclerosis (ALS).

Jung et al. presented a novel 5 time-point longitudinal PET/MRI study of MCI patients in South Korea demonstrating a mediating effect of progressive subcortical abnormality on memory and executive function. Notably, time-varying change in thalamic volume, but not subcortical vascular burden, mediated progressive cognitive dysfunction. This builds upon the growing focus of thalamic abnormality as an important imaging biomarker of early and dissociable neurodegenerative pathology (1, 2), providing much needed longitudinal evidence from the early stage of the clinical dementia continuum. The importance of thalamic integrity in neurodegenerative disorders also encompasses its extensive cortical and subcortical (i.e., striatum, basal ganglia) connections, as demonstrated by the two studies in PD. While Mi et al. report selective abnormalities in regional functional striatal-cortical connectivity associated with disease duration in idiopathic PD, Choi et al. showed altered inter-regional metabolic connectivity in the striato-thalamo-cortical and cerebello-thalamo-cortical motor circuits in dementia with Lewy body.

The clinical utility of quantitative changes involving cortical-striatal pathways appears to also be relevant in the early stages of ALS. Kocar et al. trained and validated a neural network based on 502 brain MRI scans for the detection of ALS pathology, relative to matched controls, utilizing a proposed disease staging model of white-matter propagation (3). As expected, cortico-spinal and inter-hemispheric projections, mediating upper/lower motor neuron communication, held the highest classifier weighting. Fractional anisotropy of cortico-striatal white matter, however, also demonstrated notable weighting in the classifier model. This is consistent with growing evidence from the ALS literature highlighting the association between extra-motor subcortical abnormality and functional decline, as potential secondary pathways modulating disease progression rate (3, 4).

The compiled studies show the increasing progress and sophistication applied to subcortical analyses across neurodegenerative conditions and the importance of regional subcortical abnormality as imaging targets for capturing sub-clinical pathology, clinical phenotyping, and disease progression. Despite this promising progress, several limitations still emerge which need to be addressed in future studies. Specifically, the heterogeneity of imaging methods and their application makes it difficult to compare findings across studies, even within the same disease pathophysiology. It highlights a problem which has been recognized by several international working groups (hippocampus subfield group, for details see https://hippocampalsubfields.com; thalamic nuclei group, for details see https://thalamicseg.weebly.com) that there is an important need to standardize imaging approaches toward subcortical structures. Such standardization, in the form of harmonization of acquisition and/or analysis protocols would allow more systematic investigation of subcortical changes across time and facilitate multi-center observational and intervention studies for neurodegenerative syndromes. Such standardization processes have been established for some time for cortical imaging but remain overlooked for subcortical regions.

Touching upon the impact of standardized analysis protocols, Temp et al. provide a timely perspective article introducing the unique advantages of Bayes theorem and applied parameter estimation as a methodological alternative to the more commonly applied *p* value focused Fisher statistics for detecting statistical significance. Specifically, the ability to allow for comparisons of multiple alternative hypotheses for small effects is of interest when analyzing subcortical structures, as it allows for discrimination between groups of patients or at the individual patient level. With recent neuroimaging publications of population-based brain development trajectories across the human life-span (5), it has become increasingly evident that accurate modeling of inter-individual differences is a critical step for advancing current knowledge of neurodegeneration.

Taken together, it emerges that cortical-subcortical loops and the integrity of subcortical structures are a critical aspect for further investigation, in particular for determining how subcortical changes contribute to “cortical symptomology.” There is still a common notion that subcortical changes in many neurodegenerative conditions emerge from Wallerian degeneration caused by cortical changes, based on the significant body of literature reporting cortical findings. However, clearly this is not yet established since subcortical changes might precede cortical changes for some neurodegenerative condition or certain symptoms. Better standardization and modeling of inter-individual differences will allow us to establish the relationship of cortical and subcortical changes in disease progression and symptomology across neurodegenerative conditions. Only then will it be possible to delineate cortical and subcortical contributions to neurodegenerative syndromes, without the current, inherent focus on cortical changes.

We hope the readers enjoy our Research Topic submission as it highlights an important but under-researched area, which has great potential for future neurodegenerative disease diagnosis and management.

## Author contributions

ST, JM, and MH contributed equally to the interpretation and views expressed in the editorial. All authors contributed to the article and approved the submitted version.

## Conflict of interest

The authors declare that the research was conducted in the absence of any commercial or financial relationships that could be construed as a potential conflict of interest.

## Publisher's note

All claims expressed in this article are solely those of the authors and do not necessarily represent those of their affiliated organizations, or those of the publisher, the editors and the reviewers. Any product that may be evaluated in this article, or claim that may be made by its manufacturer, is not guaranteed or endorsed by the publisher.
